# Prognostic Impact of Supranormal LVEF Following Mitral Valve TEER in Patients With Secondary Atrial MR

**DOI:** 10.1016/j.jacasi.2025.09.008

**Published:** 2025-10-29

**Authors:** Yuki Hida, Teruhiko Imamura, Shuhei Tanaka, Hiroshi Ueno, Koichiro Kinugawa, Shunsuke Kubo, Masanori Yamamoto, Mike Saji, Masahiko Asami, Yusuke Enta, Masaki Nakashima, Shinichi Shirai, Masaki Izumo, Shingo Mizuno, Yusuke Watanabe, Makoto Amaki, Kazuhisa Kodama, Junichi Yamaguchi, Yuki Izumi, Toru Naganuma, Hiroki Bota, Yohei Ohno, Masahiro Yamawaki, Kazuki Mizutani, Toshiaki Otsuka, Kentaro Hayashida, OCEAN-Mitral Investigators

**Affiliations:** a Second Department of Internal Medicine, University of Toyama, Japan; b Department of Cardiology, Kurashiki Central Hospital, Japan; c Department of Cardiology, Toyohashi Heart Center, Japan; d Department of Cardiology, Nagoya Heart Center, Japan; e Department of Cardiology, Gifu Heart Center, Japan; f Department of Cardiology, Sakakibara Heart Institute, Tokyo, Japan; g Division of Cardiovascular Medicine, Department of Internal Medicine, Toho University Faculty of Medicine, Tokyo, Japan; h Division of Cardiology, Mitsui Memorial Hospital, Tokyo, Japan; i Department of Cardiology, Sendai Kosei Hospital, Japan; j Division of Cardiology, Kokura Memorial Hospital, Kitakyushu, Japan; k Division of Cardiology, St. Marianna University School of Medicine Hospital, Kawasaki, Japan; l Department of Cardiology, Shonan Kamakura General Hospital, Kanagawa, Japan; m Department of Cardiology, Teikyo University School of Medicine, Tokyo, Japan; n Department of Cardiology, National Cerebral and Cardiovascular Center, Suita, Japan; o Division of Cardiology, Saiseikai Kumamoto Hospital Cardiovascular Center, Japan; p Department of Cardiology, Tokyo Woman’s Medical University, Japan; q Department of Cardiology, New Tokyo Hospital, Chiba, Japan; r Department of Cardiology, Sapporo Higashi Tokushukai Hospital, Japan; s Department of Cardiology, Tokai University School of Medicine, Isehara, Japan; t Department of Cardiology, Saiseikai Yokohama City Eastern Hospital, Kanagawa, Japan; u Division of Cardiology, Department of Medicine, Kindai University Faculty of Medicine, Osaka, Japan; v Department of Hygiene and Public Health, Nippon Medical School, Tokyo, Japan; w Department of Cardiology, Keio University School of Medicine, Tokyo, Japan

**Keywords:** heart failure, hemodynamics, MitraClip, structural heart diseases, valvular disease

## Abstract

**Background:**

The concept of supranormal left ventricular ejection fraction (snLVEF) has recently gained prominence. Among patients with heart failure and preserved ejection fraction, those exhibiting snLVEF have demonstrated worse clinical outcomes compared to individuals with normal left ventricular ejection fraction (LVEF). However, the prognostic implications of snLVEF in the context of transcatheter edge-to-edge repair (TEER) for secondary atrial mitral regurgitation remain insufficiently elucidated.

**Objectives:**

The purpose of this study was to investigate the prognostic impact of snLVEF in patients receiving TEER for secondary atrial mitral regurgitation.

**Methods:**

This study evaluated patients with an LVEF ≥50% who underwent TEER using the MitraClip system for secondary atrial mitral regurgitation, as documented in the multicenter, investigator-initiated OCEAN-Mitral registry. snLVEF was defined as an LVEF ≥65%. The association between baseline snLVEF and 2-year mortality following TEER was analyzed.

**Results:**

The study included 529 patients (median age 83 years, 213 men). Of these, 137 patients were identified with snLVEF, while the remainder exhibited normal LVEF. Patients with snLVEF were characterized by smaller left ventricular dimensions and more severe tricuspid regurgitation relative to those with normal LVEF. The presence of snLVEF was independently associated with a significantly elevated risk of 2-year mortality following TEER, with an adjusted HR of 1.86 (95% CI: 1.21-2.86; *P =* 0.005).

**Conclusions:**

snLVEF emerged as an independent predictor of increased mortality following TEER for secondary atrial mitral regurgitation vs normal LVEF. (OCEAN-Mitral registry; UMIN000023653)

Recent clinical guidelines categorize heart failure based on left ventricular ejection fraction (LVEF) into 3 distinct subtypes: heart failure with reduced LVEF, heart failure with mildly reduced LVEF, and heart failure with preserved left ventricular ejection fraction (HFpEF).^1^, ^2^, ^3^ These classifications are critical, because therapeutic strategies and their efficacies vary significantly depending on the LVEF category. Over recent years, the prevalence of clinically diagnosed HFpEF has been on the rise.^4^ However, unlike heart failure with reduced LVEF and those with mildly reduced LVEF, limited pharmacological interventions have demonstrated significant efficacy in reducing mortality and morbidity in patients with HFpEF.

Within the HFpEF spectrum, a subset of patients with supranormal left ventricular ejection fraction (snLVEF)—typically defined as an LVEF exceeding 65%—has garnered attention caused by their paradoxically worse clinical outcomes.^5^ Studies have indicated that patients with snLVEF exhibit higher mortality compared with those with normal left ventricular ejection fraction (nLVEF), defined as an LVEF ranging from 50% to 65%.^6^^,^^7^ Consistent with these findings, our previous research on candidates for transcatheter aortic valve implantation, a population with a higher prevalence of snLVEF, revealed less favorable outcomes among those with snLVEF compared with their nLVEF counterparts.^8^

Secondary mitral regurgitation (MR) is a well-recognized factor that exacerbates the prognosis of patients with heart failure, contributing to higher morbidity and mortality compared with those without valvular involvement.^9^ The Cardiovascular Outcomes Assessment of the MitraClip Percutaneous Therapy for heart failure patients with functional mitral regurgitation (COAPT) trial demonstrated that transcatheter edge-to-edge repair (TEER) using the MitraClip system (Abbott Vascular) significantly reduces all-cause mortality and heart failure rehospitalizations in patients with heart failure with reduced LVEF and secondary MR.^10^^,^^11^ However, there is a paucity of data regarding the outcomes of TEER in patients with preserved LVEF, particularly in those with snLVEF, given the novelty of the concept of snLVEF.

A deeper understanding of the clinical characteristics and prognostic implications of snLVEF in patients undergoing TEER for secondary MR could inform optimal patient selection and enhance periprocedural management in this cohort. Therefore, this study aims to evaluate the clinical profiles of patients with snLVEF and secondary MR and assess the prognostic impact of snLVEF compared with nLVEF in individuals undergoing TEER for secondary atrial MR.

## Methods

### Participant selection

This study was conducted retrospectively using data from the prospectively maintained multicenter, investigator-initiated OCEAN-Mitral registry.^12^ The registry includes information on patients who underwent TEER using the MitraClip system for secondary atrial MR. Patients who satisfied all the following items were assigned to secondary atrial MR: 1) left atrial enlargement (left atrial diameter ≥40 mm or left atrial volume index ≥34 mL/m^2^); 2) preserved ejection fraction (LVEF ≥50%); and 3) no left ventricular enlargement (left ventricular end-diastolic volume ≤150 mL or left ventricular end-diastolic diameter ≤55 mm). Individuals with primary MR were excluded from the analysis.

This study was registered in the University Hospital Medical Information Network Clinical Trials Registry (UMIN000023653). The research protocol for the registry database was approved by the Institutional Review Board of each participating institution, ensuring compliance with the ethical principles outlined in the Declaration of Helsinki.

### Study design

The present study was retrospectively conducted by using a prospectively constructed OCEAN-Mitral registry database. The independent variable was defined as baseline snLVEF (vs nLVEF) measured just before TEER. Here, LVEF ≥65% was assigned to snLVEF and LVEF between 50% and 65% was assigned to nLVEF. Day 0 was defined at the time of TEER using the MitraClip system. The primary outcome was defined as 2-year mortality after the procedure.

### TEER procedure

The decision to proceed with TEER was made by a multidisciplinary local heart team consisting of interventional cardiologists, general cardiologists, cardiothoracic surgeons, imaging cardiologists, medical engineers, and other experienced health care professionals. The severity of MR was quantified using the proximal isovelocity surface area method, as assessed by board-certified echocardiography experts at each participating institution. This decision-making process involved comprehensive discussions with patients and their families, ensuring that dedicated informed consent procedures were thoroughly followed.

The TEER procedure using the MitraClip system adhered to established standardized protocols. Conducted under general anesthesia, the procedure was guided by fluoroscopy and transesophageal echocardiography. Following transseptal puncture via femoral vein access, a 24-F guide catheter was introduced into the left atrium. The clip delivery system was then advanced to a position above the origin of the MR jet and subsequently navigated into the left ventricular cavity. The mitral leaflets were engaged, and the clip was temporarily closed to approximate the leaflets. If a satisfactory reduction in MR was achieved, the clip was permanently deployed. In cases where additional reduction in MR was necessary, a second clip was considered based on an assessment of the residual MR and the mean pressure gradient across the mitral valve.

### Follow-up

Patients were closely monitored during the index hospitalization for any periprocedural complications. Discharge was permitted only after confirming the absence of critical procedure-related complications. Following discharge, patients attended scheduled follow-up visits at the outpatient clinics of the respective institutions or their affiliated centers, under the care of board-certified cardiologists.

Heart failure medications were adjusted based on the patients’ symptoms and diagnostic findings obtained during these visits, with modifications made at the discretion of the attending cardiologists to ensure optimal management.

### Clinical variables obtained

Data for this study were sourced from the OCEAN-Mitral registry database.^12^ Clinical variables included baseline characteristics, comprising demographic information, comorbidities, laboratory findings, echocardiographic evaluations, and medication records. Medication data specifically included the administration of intravenous inotropes. Echocardiographic assessments were performed by experienced echocardiographers, and the measurements were validated by the board-certified echocardiologists per the guidelines established by the American Society of Echocardiography. LVEF was calculated by the modified Simpson’s method according to the guidelines. Notably, snLVEF, defined as an LVEF ≥65%, was analyzed as an independent variable.

Procedural data, such as anesthesia time, were also collected. Acute procedural success was defined as achieving moderate or less residual MR after the intervention. Postprocedural data included echocardiographic evaluations related to valvular disease, duration of hospitalization, in-hospital mortality, and adverse event documentation.

Clinical outcomes were tracked for up to 2 years following the procedure or until the conclusion of the study period. Adverse events included all-cause readmissions, single-leaflet device attachment, leaflet tear, and clip embolism. The primary outcome was 2-year all-cause mortality, with specific categorization of cardiovascular and noncardiovascular deaths. The number of heart failure-related readmissions was also recorded.

### Statistical analyses

Continuous variables were expressed as median (Q1-Q3), while categorical variables were presented as counts with corresponding percentages. The cohort was stratified into 2 groups based on baseline LVEF levels: the snLVEF group, comprising individuals with LVEF ≥65%, and the nLVEF group, comprising individuals with LVEF between 50% and 65%. Continuous variables were compared between groups using the Mann-Whitney *U* test, and categorical variables were compared using the chi-square test or Fisher exact test, as appropriate.

Time-to-event analyses were conducted to evaluate the prognostic impact of snLVEF vs nLVEF, or LVEF as a continuous variable, on 2-year mortality and other secondary outcomes. The effect of LVEF subgroups (LVEF between 55% and 60%, 60% and 65%, and ≥65%) on outcomes was assessed using Cox proportional hazards regression analysis, with the LVEF 50% to 55% group serving as the reference (the methodology of categorization was similar to the previous literature).^6^ Receiver-operating characteristics analysis was performed to identify the optimal LVEF cutoff associated with the primary outcome. Cox proportional hazards regression was further utilized to examine the impact of snLVEF on the primary outcome. Sixteen predefined potential confounding variables were included in univariable analyses alongside snLVEF. Variables with a *P* value <0.05 in univariable analysis were incorporated into the multivariable analysis using a forced entry method. Kaplan-Meier survival analysis was employed to compare 2-year mortality and the incidence of secondary outcomes between the snLVEF and nLVEF groups.

Propensity score matching was further performed between patients with and without snLVEF. The propensity score was estimated using a multivariable logistic regression model that included the variables significant in the univariable Cox proportional hazard regression analyses. A 1:2 nearest-neighbor matching without replacement was conducted using a caliper width of 0.2 of the SD of the logit of the propensity score. Covariate balance between groups before and after matching was assessed using standardized mean differences.

All statistical analyses were performed using SPSS Statistics version 23.0 software (IBM Corp). Statistical significance was determined at a 2-sided *P* value threshold of <0.05.

## Results

### Baseline characteristics of the whole cohort

Between April 2018 and June 2023, a total of 3,764 patients underwent MitraClip implantation and were enrolled in the OCEAN-Mitral registry. Of these, 1,125 patients with primary MR were excluded from the present analysis. Among the remaining 2,639 patients with secondary MR, 531 patients with secondary atrial MR were identified. Two patients with insufficient data were excluded. Finally, 529 patients with secondary atrial MR, all of whom had LVEF ≥50%, were included in this retrospective study.

Baseline characteristics are displayed in Table 1. Median age was 83 years (Q1-Q3: 79-86 years) and 213 (40%) were men. Median clinical frailty scale was 4 (Q1-Q3: 3-5). Estimated glomerular filtration rate was 34.7 mL/min/1.73 m^2^ (Q1-Q3: 25.1-45.8 mL/min/1.73 m^2^) and plasma B-type natriuretic peptide level was 265 pg/mL (Q1-Q3: 137-505 pg/mL). Median LVEF was 59% (55%, 64%) and left atrial volume index was 91.7 mL/m^2^ (Q1-Q3: 65.2-137.0 mL/m^2^).

**Table 1:** Baseline Characteristics

	Total (N = 529)	snLVEF (n = 137)	nLVEF (n = 392)	*P* Value
Demographics				
Age, y	83 (79-86)	84 (81-87)	83 (79-86)	0.049^a^
Male	213 (40)	55 (40)	158 (40)	0.53
Body surface area, m^2^	1.44 (1.34-1.58)	1.46 (1.35-1.58)	1.45 (1.34-1.59)	0.77
NYHA functional class I/II/III/IV	10/212/255/52	4/51/69/13	6/161/186/39	0.66
Clinical frailty scale	4 (3-5)	4 (3-5)	4 (3-5)	0.087
Vital signs				
Systolic blood pressure, mm Hg	110 (100-126)	112 (102-126)	108 (98-125)	0.053
Pulse rate, beats/min	71 (62-82)	68 (60-86)	71 (63-82)	0.26
Types of MR				
Primary/secondary MR	0/529	0/137	0/392	—
History of heart failure				
Heart failure duration ≥5 y	84 (16)	17 (12)	67 (17)	0.12
Heart failure admission ≥3 times	111 (21)	23 (17)	88 (22)	0.099
Heart failure admission time within a year	1 (0-2)	1 (1-2)	1 (0-2)	0.64
Comorbidity				
Hypertension	373 (71)	100 (73)	273 (70)	0.27
Dyslipidemia	229 (43)	54 (39)	175 (45)	0.17
Diabetes mellitus	114 (22)	29 (21)	85 (22)	0.98
Coronary artery disease	120 (23)	25 (18)	95 (24)	0.35
History of VT	12 (2)	2 (15*)*	10 (3)	0.46
Peripheral artery disease	39 (7)	8 (6)	31 (8*)*	0.28
Inotropes infusion use	26 (5)	6 (4)	20 (5)	0.47
Scores				
EuroSCORE II	4.17 (2.88-6.49)	4.39 (3.24-7.12)	4.08 (2.85-6.12)	0.91
STS score for mitral valve repair	6.7 (4.0-10.0)	6.9 (4.5-9.1)	6.4 (3.8-10.0)	0.71
STS score for mitral valve replacement	9.1 (6.1-12.9)	9.7 (8.2-12.8)	8.8 (6.0-13.0)	0.18
Laboratory data				
Hemoglobin, g/dL	11.2 (10.0-12.5)	10.7 (9.5-11.8)	11.2 (10.2-12.6)	0.037^a^
Serum albumin, g/dL	3.7 (3.4-4.0)	3.5 (3.4-3.8)	3.7 (3.4-4.0)	0.07
Serum sodium, mEq/L	140 (138-141)	140 (138-142)	139 (138-142)	0.42
eGFR, mL/min/1.73 m^2^	34.7 (25.1-45.8)	33.8 (23.8-45.2)	34.9 (25.2-46.1)	0.69
Plasma BNP, pg/mL	265 (137-505)	256 (138-523)	267 (147-498)	0.69
Electrocardiogram data				
Atrial fibrillation	439 (83)	111 (81)	328 (84)	0.28
QRS width, ms	100 (90-121)	98 (91-110)	102 (90-125)	0.29
Echocardiography data				
LVDD, mm	50 (45-54)	48 (43-52)	50 (46-55)	0.002^a^
LVEDV, mL	93 (70-115)	79 (63-103)	96 (76-118)	0.002^a^
LVEF, %	59 (55-64)	68 (67-70)	57 (53-61)	<0.001^a^
LAV index, mL/m^2^	91.7 (65.2-137.0)	90.4 (65.7-126.9)	91.9 (65.3-140.3)	0.89
Moderate or greater AR	53 (10)	14 (10)	39 (10)	0.52
Moderate or greater TR	266 (50)	80 (58)	186 (47)	0.017^a^
Moderate or greater AR and TR	29 (5)	8 (6)	21 (5)	0.49
MR effective regurgitant orifice area, cm^2^	0.30 (0.21-0.39)	0.31 (0.23-0.41)	0.30 (0.21-0.37)	1.0
E/e' ratio (average)	13.7 (10.5-18.0)	12.8 (9.6-18.3)	13.6 (10.6-18.0)	0.84
Tricuspid annular plane excursion, mm	16.2 (13.5-19.2)	16.5 (13.5-20.1)	16.0 (13.0-19.0)	0.13
TRPG, mm Hg	30 (24-41)	31 (25-41)	31 (24-42)	0.31
Medication use				
Beta-blockers	361 (68)	90 (66)	271 (69)	0.38
Renin-angiotensin system inhibitors	318 (60)	91 (66)	227 (58)	0.08
Mineralocorticoid receptor antagonists	259 (49)	72 (53)	187 (48)	0.33
SGLT2 inhibitors	79 (15)	24 (18)	55 (14)	0.20
Diuretic agents	443 (84)	118 (86)	325 (83)	0.23

Values are median (Q1-Q3) and compared between the 2 groups by Mann-Whitney *U* test or n (%) and compared between the 2 groups by chi-square test or Fisher exact test.

AR = aortic regurgitation; BNP = B-type natriuretic peptide; eGFR = estimated glomerular filtration rate; LAV = left atrial volume; LVDD = left ventricular end-diastolic diameter; LVEDV = left ventricular end-diastolic volume; MR = mitral regurgitation; nLVEF = normal left ventricular ejection fraction; SGLT2 = sodium-glucose cotransporter 2; snLVEF = supranormal left ventricular ejection fraction; TR = tricuspid regurgitation; VT = ventricular tachycardia.

a *P <* 0.05.

### Clinical features of snLVEF

**Figure 1:**
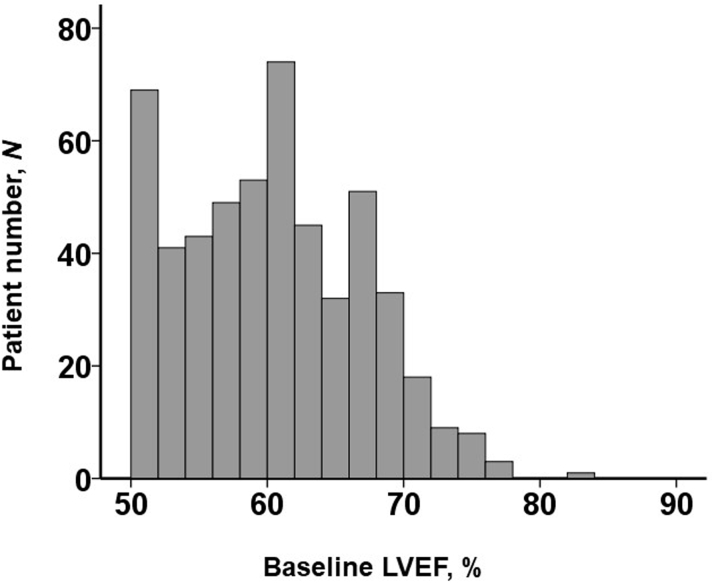
Distribution of Baseline LVEF All patients had left ventricular ejection fraction (LVEF) ≥50% according to the study protocol. Of them, 137 patients (26%) had LVEF ≥65%, which was assigned to supranormal LVEF (an independent variable).

The distribution of baseline LVEF is displayed in Figure 1. Of all, 137 patients (26%) had snLVEF, which was defined as LVEF ≥65%. A comparison of baseline characteristics between those with snLVEF vs nLVEF was stated in Table 1. Baseline characteristics were mostly not significantly different between the 2 groups, except for several variables. Patients in the snLVEF were older and had more advanced anemia, a smaller left ventricle, and a higher prevalence of moderate or greater tricuspid regurgitation (*P <* 0.05 for all).

When patients were divided into 4 subgroups by baseline LVEF (LVEF between 50% and 55% [n = 122], LVEF between 55% and 60% [n = 133], LVEF between 60% and 65% [n = 133], and LVEF above 65% [n = 103]), the prevalence of atrial fibrillation and left atrial volume index were not significantly stratified across LVEF categories *(P =* 0.23 and *P =* 0.27, respectively). The prevalence of moderate or greater tricuspid regurgitation was significantly stratified by LVEF categories *(P =* 0.029).

### Periprocedural data

Most patients (95%) achieved the acute procedural success, indicating moderate or less residual MR (Table 2). Anesthesia time was 153 minutes (Q1-Q3: 125-191 minutes) and procedural time was 80 minutes (Q1-Q3: 58-105 minutes). The periprocedural data did not differ between those with snLVEF and those with nLVEF (*P >*0.05 for all).

**Table 2:** Periprocedural Data

	Total (N = 529)	snLVEF (n = 137)	nLVEF (n = 392)	*P* Value
Procedural data				
Anesthesia time, min	153 (125-191)	149 (131-201)	155 (123-183)	0.67
Procedural time, min	80 (58-105)	81 (61-106)	78 (58-105)	0.41
Device time, min	50 (35-73)	44 (32-69)	50 (38-74)	0.29
Fluoroscopy duration, min	22 (15-32)	22 (14-34)	23 (15-32)	0.29
MV mean gradient after clip, mm Hg	2.3 (2.0-3.3)	2.9 (2.0-3.0)	2.3 (2.0-3.8)	0.082
LAA emptying velocity, cm/s	18 (12-30)	17 (13-26)	18 (11-31)	0.87
Acute procedural success	504 (95)	128 (93)	376 (96)	0.17
Post-procedural event data				
Pericardial effusion	17 (3)	7 (5)	10 (3)	0.14
Access site complication	17 (3)	3 (2)	14 (4)	0.43
TEE-associated complication	7 (1)	3 (2)	4 (1)	0.30
Pulmonary complication	4 (1)	0 (0)	4 (1)	0.24
Acute kidney injury	12 (2)	4 (3)	8 (2)	0.55
In-hospital death	6 (1)	2 (1)	4 (1)	0.50
Home discharge	394 (75)	102 (74)	292 (74)	0.39

Values are median (Q1-Q3) and compared between the 2 groups by Mann-Whitney *U* test or n (%) and percentages and compared between the 2 groups by chi-square test or Fisher exact test.

LAA = left atrial appendage; MV = mitral valve; TEE = transesophageal echocardiography; other abbreviations as in Table 1.

Some patients encountered postprocedural events (Table 2). Notably, 6 patients deceased during the index hospitalization. The postprocedural event rates were not significantly different between the 2 groups (*P >* 0.05). In total, 394 patients were discharged to their homes. Its prevalence was not significantly different between the 2 groups *(P =* 0.39). Among 168 patients who did not have atrial fibrillation before TEER, the incidence of de novo atrial fibrillation following TEER tended to be higher in patients with snLVEF than those with nLVEF (18% vs 11%; *P =* 0.16).

### Postdischarge events

Adverse event rates are displayed in Table 3. There were no significant differences in the incidence of these events between those with snLVEF and those with nLVEF (*P >* 0.05 for all). The incidence of recurrent MR was not significantly different between the 2 groups *(P =* 0.26).

**Table 3:** Postdischarge Clinical Events

	snLVEF (n = 137)	nLVEF (n = 392)	*P* Value
All-cause readmission	51 (37)	167 (43)	0.16
Single-leaflet device attachment	3 (2)	4 (1)	0.30
Leaflet tear	3 (2)	5 (1)	0.45
Clip embolism	0 (0)	0 (0)	—
Recurrence of moderate-to-severe or severe MR	2 (1)	13 (3)	0.26

Values are n (%) and percentages and compared between the 2 groups by chi-square test or Fisher exact test.

Abbreviations as in Table 1.

### Trajectory of echocardiography parameters

The trajectory of echocardiography parameters in snLVEF group and nLVEF group is displayed in Table 4, respectively. Most parameters improved following TEER, irrespective of the values of baseline LVEF. Notably, left atrial volume index significantly decreased in the nLVEF group (*P <* 0.001) but remained unchanged in the snLVEF group *(P =* 0.27).

**Table 4:** Trajectory of Echocardiography Parameters Following TEER

	Baseline	Discharge	1 Month	1 Year	*P* Value
snLVEF (n = 137)					
LVEDD, mm	48 (43-52)	44 (42-47)	45 (39-49)	48 (41-53)	<0.001^a^
LVEDV, mL	79 (63-103)	70 (58-96)	70 (57-96)	72 (58-100)	<0.001^a^
LVEF, %	68 (67-70)	62 (57-67)	63 (59-66)	62 (57-65)	<0.001^a^
LAVI, mL/m^2^	90.4 (65.7-126.9)	73.3 (55.8-130.5)	78.7 (59.0-129.2)	85.4 (67.5-154.4)	0.27
E/e' ratio (average)	12.8 (9.6-18.3)	23.8 (17.5-27.3)	25.9 (22.3-32.4)	23.6 (17.7-30.2)	<0.001^a^
TRPG, mm Hg	31 (25-41)	29 (25-36)	30 (25-35)	33 (28-44)	0.15
Significant MR	117/20	5/130	5/109	3/77	<0.001^a^
Plasma BNP, pg/mL	256 (138-523)	—	196 (148-767)	158 (85-272)	0.001^a^
nLVEF (n = 392)					
LVEDD, mm	50 (46-55)	48 (44-54)	48 (44-53)	48 (32-53)	<0.001^a^
LVEDV, mL	96 (76-118)	87 (71-109)	92 (69-107)	83 (64-107)	<0.001^a^
LVEF, %	57 (53-61)	55 (49-59)	56 (51-61)	57 (51-63)	<0.001^a^
LAV index, mL/m^2^	91.9 (65.3-140.3)	88.2 (62.6-134.5)	82.7 (70.4-139.8)	82.2 (62.9-119.4)	<0.001^a^
E/e' ratio (average)	13.6 (10.6-18.0)	20.4 (16.4-28.1)	22.8 (18.9-29.8)	25.0 (19.6-30.2)	<0.001^a^
TRPG, mm Hg	31 (24-42)	29 (24-36)	29 (24-35)	29 (24-35)	0.12
Significant MR	324/68	9/380	7/307	9/223	<0.001^a^
Plasma BNP, pg/mL	267 (147-498)	—	190 (130-492)	116 (43-289)	<0.001^a^

Values are median (Q1-Q3) and their trends were assessed by the Friedman test or n (%) and their trends were assessed by the Cochran Q test. Parameters were obtained at baseline before TEER, at index discharge, 1 month, and 1 year after TEER.

TRPG = tricuspid regurgitation pressure gradient; other abbreviations as in Table 1.

a *P <* 0.05. Significant MR was defined as moderate-to-severe or severe MR.

### Two-year prognostic impact of baseline LVEF

**Figure 2:**
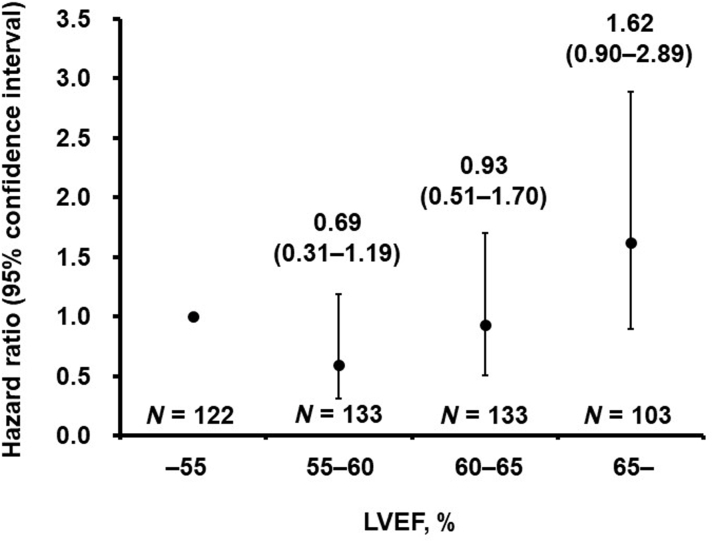
HR of Each LVEF Subgroup for the Primary Outcome Patients were assigned to 4 subgroups according to their baseline left ventricular ejection fraction (LVEF): LVEF <55%, LVEF between 55% and 60%, LVEF between 60% and 65%, and LVEF ≥65%. The group with LVEF <55% was defined as a reference. Only the group with LVEF ≥65% had an HR above 1.00.

During the 2-year observation period after the procedure, 89 patients were deceased (the primary outcome). First, patients were categorized into 4 groups according to their baseline LVEF levels as stated in the previous text. The HR of each subgroup for the primary outcome are displayed in Figure 2. When the HR of individuals with LVEF <55% was defined as a reference, the HR in the group of LVEF above 65% was highest: 1.62 (95% CI: 0.90**-**2.89), although its difference did not reach statistical significance. Notably, the HRs of LVEF between 55% and 60% and between 60% and 65% were both below 1.0.

**Figure 3:**
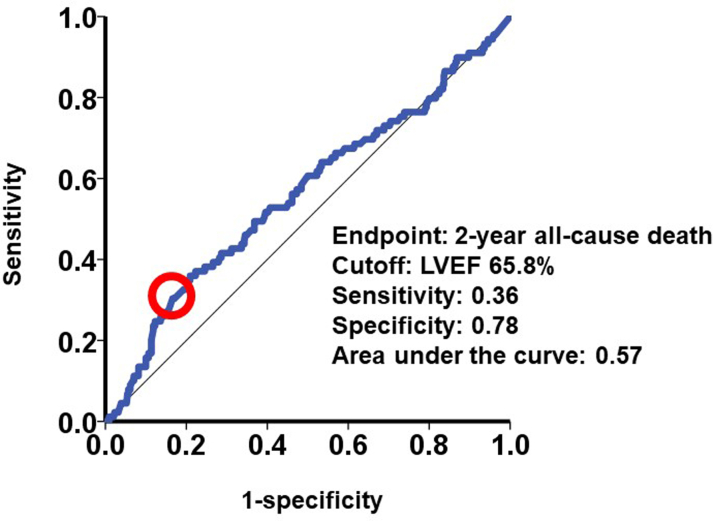
Receiver-Operating Characteristic Analysis for the 2-Year Mortality A statistical cutoff to predict the primary outcome was calculated as 65.8% of left ventricular ejection fraction (LVEF). This is a rationale why we adopted the cutoff of LVEF 65% in the present study.

When LVEF was assumed as a continuous variable, a cutoff of LVEF for the primary outcome was calculated as 65.8% with a sensitivity of 0.36, specificity of 0.78, and the area under the curve of 0.57 (Figure 3). Thus, snLVEF was defined as LVEF above 65% in the present study (others were assigned to nLVEF).

### Two-year prognostic impact of baseline snLVEF (vs nLVEF)

A total of 17 predefined potential variables were included in the univariable analysis to assess their prognostic impact on the primary outcome, including snLVEF (Table 5). The proportional hazards assumption was validated for each covariate and the overall model using Schoenfeld residuals before applying the Cox model, and no relevant violations were observed.

**Table 5:** Impact of Potential Baseline Variables on the 2-Year Mortality

	Univariable Analysis		Multivariable Analysis	
	HR (95% CI)	*P* Value	HR (95% CI)	*P* Value
Age, y	1.04 (1.00-1.08)	0.042^a^	0.99 (0.96-1.03)	0.62
Male	1.34 (0.89-2.04)	0.17		
Clinical frailty scale	1.40 (1.21-1.62)	<0.001^a^	1.22 (1.03-1.44)	0.021^a^
HF admission ≥3 times	2.07 (1.34-3.20)	0.001^a^	1.57 (0.99-2.50)	0.055
Atrial fibrillation	0.76 (0.46-1.25)	0.27		
Inotropes infusion	3.35 (1.68-6.68)	0.001^a^	1.34 (0.63-2.85)	0.44
Hemoglobin, g/dL	0.86 (0.76-0.98)	0.027^a^	1.05 (0.91-1.21)	0.53
Serum albumin, g/dL	0.28 (0.19-0.42)	<0.001^a^	0.34 (0.21-0.54)	<0.001^a^
eGFR, mL/min/1.73 m^2^	0.99 (0.98-1.01)	0.39		
Plasma BNP, pg/mL	1.00 (1.00-1.01)	0.003^a^	1.00 (1.00-1.01)	0.11
LVEDV, mL	0.99 (0.98-1.01)	0.26		
LAV index, mL/m^2^	1.01 (1.00-1.01)	0.11		
snLVEF versus nLVEF	1.86 (1.21-2.86)	0.005^a^	1.84 (1.12-2.93)	0.010^a^
Moderate or greater TR	1.53 (1.00-2.34)	0.049^a^	1.53 (0.98-2.38)	0.063
RAS inhibitors use	0.78 (0.52-1.19)	0.25		
MRA use	1.47 (0.96-2.25)	0.074		
SGLT2 inhibitor use	1.23 (0.67-2.27)	0.50		

Cox proportional HR regression analysis was performed to investigate the prognostic impact of the potential variables on the 2-year mortality, including snLVEF. Potential variables were included in the univariable analyses. Variables with *P <* 0.05 in the univariable analyses were included in the multivariable analysis with a forced entry method for the adjustment.

eGFR = estimated glomerular filtration rate; HF = heart failure; MRA = mineralocorticoid receptor antagonist; RAS = renin-angiotensin system; other abbreviations as in Table 1.

a *P <* 0.05.

After adjusting for 8 variables that demonstrated statistical significance (*P <* 0.05) in the univariable analysis, snLVEF remained an independent predictor of the primary outcome, with a HR of 1.84 (95% CI: 1.12-2.93; *P =* 0.010). We confirmed that all variance inflation factors of each variable included in the multivariable analysis were within 5.0. The prognostic impact of snLVEF remained significant with an HR of 1.88 (95% CI: 1.13-2.96; *P =* 0.011) when we included atrial fibrillation and left atrial volume index in another multivariable model, despite their nonsignificant prognostic impact in the univariable analysis.

**Figure 4:**
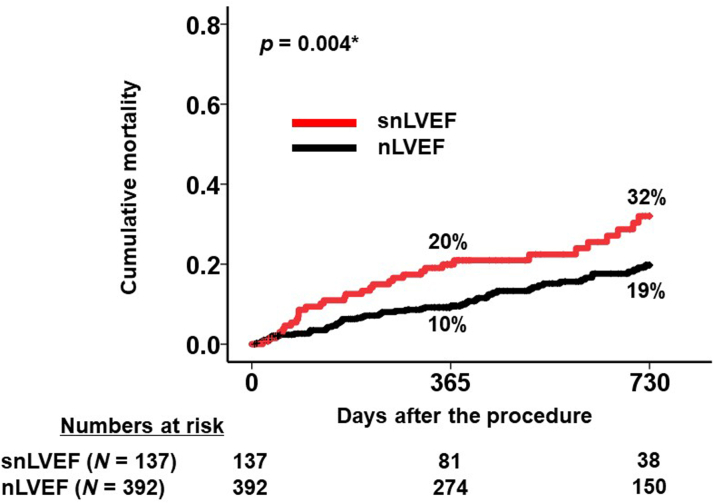
2-Year Cumulative Mortality Between snLVEF and nLVEF Groups Patients with baseline supranormal left ventricular ejection fraction (snLVEF) had significantly higher cumulative mortality during the 2-year observational period following the procedure. snLVEF was defined as left ventricular ejection fraction ≥65%; normal left ventricular ejection fraction (nLVEF) was defined as left ventricular ejection fraction between 50% and 65%. ∗*P <* 0.05 by the log-rank test.

Patients with snLVEF exhibited significantly higher cumulative mortality during the 2-year follow-up period compared with those with nLVEF (32% vs 19%; *P =* 0.004) (Figure 4). Among the 89 total deaths, 50 were of cardiovascular origin and 39 were noncardiovascular. snLVEF was significantly associated with cardiovascular mortality, with an HR of 2.10 (95% CI: 1.19-3.70; *P =* 0.010). However, the association between snLVEF and noncardiovascular mortality did not reach statistical significance, with an HR of 1.60 (95% CI: 0.91-2.82; *P =* 0.10).

A total of 86 patients experienced heart failure–related hospitalizations during the follow-up period. snLVEF did not significantly impact the risk of heart failure readmissions, as indicated by an HR of 1.08 (95% CI: 0.71-1.66; *P =* 0.72).

### Propensity score matching as a subanalysis

**Figure 5:**
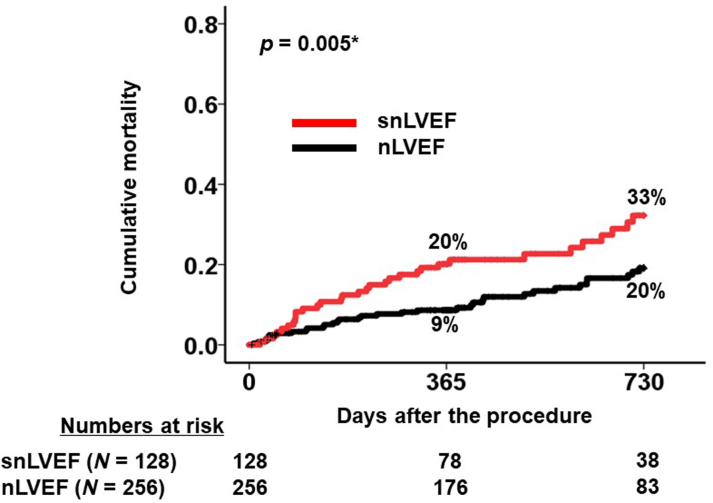
Two-Year Cumulative Mortality in the Background-Matched Cohorts Patients with baseline snLVEF had significantly higher cumulative mortality during 2-year observational period following the procedure also in the background-matched cohort. snLVEF was defined as left ventricular ejection fraction ≥65%; nLVEF was defined as left ventricular ejection fraction between 50% and 65%. ∗*P <* 0.05 by the log-rank test. Abbreviations as in Figure 4.

Propensity score matching was performed with the covariates that were significant in the univariable Cox HR regression analyses (Table 5), and 1:2 background-matched cohorts were constructed (n = 384) (Supplemental Table 1). Standardized mean differences of the utilized covariates were all within 0.1. During the 2-year observation period, 66 patients encountered the primary outcome. Cumulative mortality was significantly higher in patients with snLVEF than their counterparts (33% vs 20%; *P =* 0.005) (Figure 5).

## Discussion

This retrospective analysis, based on the multicenter, prospectively maintained OCEAN-Mitral registry data set,^12^ evaluated the prognostic impact of baseline snLVEF compared with nLVEF on 2-year mortality following TEER in patients with secondary atrial MR. For this study, snLVEF was defined as LVEF ≥65%, and nLVEF as LVEF between 50% and 65%, in alignment with existing literature and the statistical findings from this study.^5^^,^^6^

Baseline LVEF demonstrated a wide distribution, with 137 patients (26%) classified as having snLVEF. Although most baseline characteristics were comparable between the snLVEF and nLVEF groups, the snLVEF group was characterized by older age, more severe anemia, and a smaller LV size. Following TEER, most echocardiography parameters improved irrespective of the values of baseline LVEF, except for left atrial volume index, which remained unchanged in the snLVEF group.

When stratifying patients into 4 LVEF categories (50%-55%, 55%-60%, 60%-65%, and ≥65%), the HR for the primary outcome was highest in the LVEF ≥65% group (ie, snLVEF group), using the LVEF 50% to 55% group as the reference. In multivariable analysis, snLVEF was independently associated with the primary outcome, underscoring its role as a prognostic factor. Furthermore, individuals with snLVEF exhibited significantly higher 2-year mortality rates compared to those with nLVEF, highlighting the adverse prognostic implications of snLVEF in this population (Central Illustration).

**Central Illustration:**
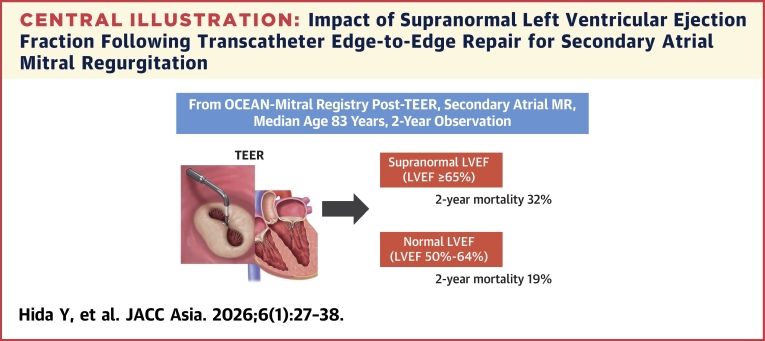
Impact of Supranormal Left Ventricular Ejection Fraction Following Transcatheter Edge-to-Edge Repair for Secondary Atrial Mitral Regurgitation Patients with supranormal left ventricular ejection fraction (LVEF) (LVEF ≥65%) at baseline had higher mortality than those with normal LVEF (LVEF between 50% and 64%) during 2-year observation period following transcatheter edge-to-edge repair (TEER). MR = mitral regurgitation.

### The Concept of snLVEF

The concept of snLVEF has garnered increasing attention in recent years. Traditionally, heart failure patients with an LVEF ≥50% have been classified under the category of HFpEF. However, emerging evidence suggests a U-shaped relationship between LVEF and clinical outcomes in this population, although this concept has yet to be fully validated.

Wehner et al^7^ identified that individuals with an LVEF exceeding 60% to 65% exhibited worse clinical outcomes compared with those with LVEF below this range. Similarly, a study by Horiuchi et al^6^ utilized a 65% threshold to define heart failure with snLVEF. In alignment with these findings, the present study adopted the same 65% cutoff to define snLVEF. Notably, we statistically validated this threshold within the context of TEER candidates, reinforcing its clinical applicability.

Following correction of MR, patients with snLVEF may exhibit physiological characteristics akin to those observed in heart failure cohorts investigated in prior studies, further supporting the relevance of this classification in understanding and managing such patients.

### Unique feature of patients with secondary atrial MR and snLVEF

Secondary MR can generally be categorized into ventricular MR and atrial MR.^13^ In patients with an LVEF <50%, ventricular MR is typically driven by systolic heart failure, resulting in mitral valve tethering caused by LV remodeling. Conversely, individuals with significant MR and preserved LVEF (≥50%)—the focus of the present study—are generally classified as having atrial MR. A key mechanism underlying atrial MR is left atrial remodeling, which leads to progressive atrial dilation and subsequent enlargement of the mitral annulus.^13^

Patients with atrial MR typically exhibit small LV dimensions accompanied by diastolic dysfunction,^14^ a feature that aligns with the unique profile of heart failure patients with snLVEF.^15^ Recent study demonstrated that the presence of concomitant heart failure with preserved LVEF was common in patients with atrial MR and associated with worse clinical outcomes.^16^ In our cohort, the snLVEF group consistently exhibited smaller LV dimensions, further supporting the notion that these patients may represent a more distinct subgroup within the atrial MR population.

In addition to smaller LV size, the snLVEF cohort demonstrated characteristics consistent with findings from previous literature, including advanced age and more pronounced anemia, although the absolute values of these differences were modest.^6^^,^^7^ Although unstable hemodynamic states, such as sepsis or hypovolemia, can also contribute to snLVEF,^7^ all patients in our cohort were hemodynamically stabilized before undergoing TEER.

### Prognostic impact of snLVEF in patients receiving TEER

In this study, the presence of snLVEF was independently associated with higher mortality following TEER compared with nLVEF. One plausible explanation for this finding is the association of snLVEF with reduced stroke volume. Significant MR reduces afterload by allowing blood to flow back into the low-resistance left atrium, leading to an overestimated LVEF despite impaired forward output. Also, left atrial dilation and reduced compliance—common in atrial MR—can impair LV filling and exacerbate diastolic dysfunction, particularly in patients with small, concentrically remodeled ventricles. Previous studies consistently demonstrated that elevated LVEF correlates with lower stroke volume, smaller LV chamber size, and more pronounced concentric remodeling.^17^ Such remodeling exacerbates diastolic dysfunction,^18^, ^19^, ^20^ potentially limiting the hemodynamic benefits of the modest increase in cardiac output observed following TEER.

Moreover, snLVEF has been linked to microvascular dysfunction, which may result in hypercontractile LV function, increased myocardial oxygen consumption, and heightened activation of the sympathetic nervous system.^21^ These pathophysiological mechanisms could contribute to the persistently elevated left atrial volume index and poorer prognosis observed in patients with snLVEF undergoing TEER.

Despite statistical adjustments, it is also possible that subclinical conditions associated with snLVEF, such as anemia and hyperthyroidism, may have further influenced the observed outcomes.^7^ These factors underscore the complexity of the prognostic landscape in this population and highlight the need for a nuanced approach to risk stratification and management in this cohort.

### Clinical implication of our findings

The independent association of snLVEF with higher mortality following TEER highlights the need for enhanced risk stratification in this population. Although TEER has demonstrated substantial benefits in patients with secondary MR and reduced LVEF, our findings suggest that the prognostic implications of snLVEF warrant careful consideration when selecting candidates for the procedure. Future clinical algorithms may benefit from incorporating LVEF as a stratifying variable to identify high-risk patients within the preserved LVEF cohort.

Given the higher mortality risk in the snLVEF cohort, individualized post-TEER management strategies are essential. This could involve closer monitoring for heart failure progression, optimizing diastolic function by adjusting hemodynamics, and addressing potential contributors to adverse outcomes such as anemia. Multidisciplinary care involving heart failure specialists, interventional cardiologists, and imaging experts may enhance long-term outcomes in this subgroup. Notably, optimal therapeutic strategy for individuals with heart failure and snLVEF remains unestablished.

### Study limitations

Although it utilized data from the largest multicenter registry in Japan, the sample size was moderate because of the strict inclusion criteria employed. Due to the small sample size, we decided to assume all continuous variables as nonparametric variables and present them as medians (Q1-Q3) to maintain statistical robustness. The TEER procedure was not randomized, and its indication was determined by the heart valve teams at each participating institution, leaving room for potential variability in patient selection. We should note that we did not include all patients with secondary atrial MR. We included only the candidates for TEER among them.

Although LVEF was comprehensively assessed using the standardized modified Simpson’s method and all included institutions were large-scale centers with board-certified echocardiologists, no core laboratory was established for centralized echocardiographic assessment, which may have introduced interinstitutional variability in measurements, particularly relevant given our use of 5% LVEF increase.

When LVEF was dealt as a binary variable (ie, snLVEF vs nLVEF), snLVEF had a significant prognostic impact. Conversely, the prognostic impact of snLVEF did not reach statistical significance when compared with a subgroup of LVEF 50% to 55% (reference group). This discrepancy would be explained by the relatively lower HR of other subgroups: LVEF 50% to 55% and LVEF 55% to 60% compared with a reference group.

Another limitation is a low area under the curve (AUC: 0.57), suggesting limited discriminatory power of LVEF alone. The 65% cutoff should therefore be interpreted cautiously, and its prognostic relevance is supported mainly by the consistent associations observed in multivariable and propensity score–matched analyses.

## Conclusions

In this retrospective analysis of a multicenter registry, snLVEF was identified as an independent predictor of higher 2-year mortality in patients with secondary atrial MR undergoing TEER. These findings highlight the prognostic importance of baseline LVEF stratification in this population, suggesting that snLVEF represents a high-risk phenotype that warrants tailored clinical management and careful patient selection for TEER. Future research is needed to elucidate the mechanisms underlying the adverse outcomes associated with snLVEF and to explore strategies to improve outcomes in this subgroup.

## Funding Support and Author Disclosures

The OCEAN-Mitral registry, which is part of the OCEAN-SHD registry, is supported by Edwards Lifesciences, Medtronic Japan, Boston Scientific, Abbott Medical Japan, and Daiichi-Sankyo Company. Drs Kubo, Maruo, Saji, Izumo, Watanabe, Amaki, Izumi, Enta, Shirai, Mizuno, Bota, Mizutani, Ueno, Yamamoto, and Hayashida are clinical proctors of transcatheter edge-to-edge repair for Abbott Medical; and have received speaker fees from Abbott Medical. Drs Asami and Kodama received speaker fees from Abbott Medical. Dr Yamaguchi is the clinical proctor of transcatheter edge-to-edge repair for Abbott Medical; and has received a lecture fee and scholarship donation from Abbott Medical. Dr Ohno has received consultant, advisor, and speaker fees from Abbott Medical. All other authors have reported that they have no relationships relevant to the contents of this paper to disclose.
