# Supplementary material for: Prognostic Impact of Supranormal LVEF Following Mitral Valve TEER in Patients With Secondary Atrial MR
Source: JACC Asia. 2025 Oct 29;6(1):27–38. doi: 10.1016/j.jacasi.2025.09.008 (PMC12833598; doi:10.1016/j.jacasi.2025.09.008)
Supplement: Supplemental Table 1 [file mmc1.docx]

**Supplementarl Table 1. Matched baseline characteristics**

|  |  |  |  |
| --- | --- | --- | --- |
|  | **snLVEF**  **(*N* = 128)** | **nLVEF**  **(*N* = 256)** | ***p*-value** |
| Demographics |  |  |  |
| Age, years | 85 (82, 87) | 83 (81, 87) | 0.87 |
| Male sex | 52 (41%) | 97 (38%) | 0.39 |
| Body surface area, m^2^ | 1.43 (1.33, 1.58) | 1.43 (1.33, 1.56) | 0.61 |
| NYHA class I/II/III/IV | 4/46/65/13 | 1/107/122/22 | 0.11 |
| Clinical frailty scale | 4 (3, 5) | 4 (3, 5) | 0.14 |
| Vital signs |  |  |  |
| Systolic blood pressure, mmHg | 118 (105, 127) | 116 (97, 124) | 0.084 |
| Pulse rate, bpm | 71 (64, 89) | 71 (63, 81) | 0.72 |
| Types of MR |  |  |  |
| Primary/secondary MR | 0/128 | 0/256 | - |
| History of heart failure |  |  |  |
| Heart failure duration ≥5 years | 17 (13%) | 46 (18%) | 0.14 |
| Heart failure admission ≥3 times | 23 (18%) | 40 (16%) | 0.35 |
| Heart failure admission time within a year | 1 (1, 2) | 1 (0, 2) | 0.37 |
| Comorbidity |  |  |  |
| Hypertension | 93 (73%) | 182 (71%) | 0.52 |
| Dyslipidemia | 52 (41%) | 117 (46%) | 0.17 |
| Diabetes mellitus | 28 (22%) | 58 (23%) | 0.86 |
| Coronary artery disease | 24 (19%) | 63 (25%) | 0.36 |
| History of VT | 2 (2%) | 7 (3%) | 0.37 |
| Peripheral artery disease | 8 (6%) | 17 (7%) | 0.52 |
| Inotropes infusion use | 5 (4%) | 9 (4%) | 0.54 |
| Scores |  |  |  |
| EuroSCORE II | 4.4 (3.1, 6.3) | 4.2 (3.2, 6.0) | 0.99 |
| STS score for mitral valve repair | 8.0 (5.5, 13.0) | 7.7 (4.7, 10.0) | 0.58 |
| STS score for mitral valve replacement | 9.6 (8.2, 14.8) | 9.2 (6.8, 13.5) | 0.95 |
| Laboratory data |  |  |  |
| Hemoglobin, g/dL | 10.7 (9.7, 12.1) | 11.1 (9.9, 12.2) | 0.92 |
| Serum albumin, g/dL | 3.5 (3.3, 3.8) | 3.7 (3.4, 4.0) | 0.18 |
| Serum sodium, mEq/L | 140 (138, 142) | 139 (138, 142) | 0.97 |
| eGFR, mL/min/1.73m^2^ | 32.2 (23.6, 47.2) | 33.1 (24.6, 45.9) | 0.82 |
| Plasma BNP, pg/mL | 332 (149, 581) | 287 (136, 557) | 0.75 |
| Electrocardiogram data |  |  |  |
| Atrial fibrillation | 102 (80%) | 204 (80%) | 0.76 |
| QRS width, msec | 98 (91, 110) | 102 (90, 123) | 0.85 |
| Echocardiography data |  |  |  |
| LVDD, mm | 47 (43, 51) | 49 (46, 54) | 0.084 |
| LVEDV, mL | 68 (57, 95) | 95 (75, 115) | 0.015* |
| LVEF, % | 69 (67, 71) | 58 (53, 62) | <0.001* |
| LAV index, mL/m^2^ | 82.6 (58.5, 133.1) | 88.6 (64.6, 130.6) | 0.70 |
| Moderate or greater AR | 14 (11%) | 27 (11%) | 0.54 |
| Moderate or greater TR | 71 (55%) | 138 (54%) | 0.49 |
| Moderate or greater AR and TR | 8 (6%) | 15 (6%) | 0.54 |
| MR effective regurgitant orifice area, cm^2^ | 0.30 (0.22, 0.41) | 0.31 (0.20, 0.38) | 0.11 |
| E/e' ratio (average) | 13.2 (10.3, 18.0) | 13.9 (11.2, 18.2) | 0.68 |
| Tricuspid annular plane excursion, mm | 16.0 (13.7, 19.4) | 16.0 (13.4, 19.3) | 0.67 |
| TRPG, mmHg | 30.5 (23.5, 39.5) | 33.0 (24.5, 42.5) | 0.17 |
| Medication use |  |  |  |
| Beta-blockers | 84 (66%) | 172 (67%) | 0.30 |
| Renin-angiotensin system inhibitors | 84 (66%) | 138 (54%) | 0.076 |
| Mineralocorticoid receptor antagonists | 69 (53%) | 116 (45%) | 0.089 |
| SGLT2 inhibitors | 23 (18%) | 34 (13%) | 0.16 |
| Diuretics | 111 (87%) | 213 (83%) | 0.34 |

Continuous variables were displayed as median (25% interquartile, 75% interquartile) and compared between the two groups by Mann-Whitney U test. Categorical variables were displayed as counts and percentages and compared between the two groups by Chi-square test or Fischer’s exact test. snLVEF, supra-normal left ventricular ejection fraction; nLVEF, normal left ventricular ejection fraction; NYHA, New York Heart Association; MR, mitral regurgitation; VT, ventricular tachycardia; eGFR, estimated glomerular filtration rate; BNP, B-type natriuretic peptide; LVDD, left ventricular end-diastolic diameter; LVEDV, left ventricular end-diastolic volume; LAV, left atrial volume; AR, aortic regurgitation; TR, tricuspid regurgitation; SGLT2, sodium-glucose co-transporter 2. *p <0.05.
